# Machine Learning Approaches Identify Genes Containing Spatial Information From Single-Cell Transcriptomics Data

**DOI:** 10.3389/fgene.2020.612840

**Published:** 2021-02-01

**Authors:** Phillipe Loher, Nestoras Karathanasis

**Affiliations:** Computational Medicine Center, Thomas Jefferson University, Philadelphia, PA, United States

**Keywords:** single cell sequencing, machine learning, feature selection, LASSO, neural networks, scRNA-seq, *Drosophila*, zebrafish

## Abstract

The development of single-cell sequencing technologies has allowed researchers to gain important new knowledge about the expression profile of genes in thousands of individual cells of a model organism or tissue. A common disadvantage of this technology is the loss of the three-dimensional (3-D) structure of the cells. Consequently, the Dialogue on Reverse Engineering Assessment and Methods (DREAM) organized the Single-Cell Transcriptomics Challenge, in which we participated, with the aim to address the following two problems: (a) to identify the top 60, 40, and 20 genes of the *Drosophila melanogaster* embryo that contain the most spatial information and (b) to reconstruct the 3-D arrangement of the embryo using information from those genes. We developed two independent techniques, leveraging machine learning models from least absolute shrinkage and selection operator (Lasso) and deep neural networks (NNs), which are applied to high-dimensional single-cell sequencing data in order to accurately identify genes that contain spatial information. Our first technique, Lasso.TopX, utilizes the Lasso and ranking statistics and allows a user to define a specific number of features they are interested in. The NN approach utilizes weak supervision for linear regression to accommodate for uncertain or probabilistic training labels. We show, individually for both techniques, that we are able to identify important, stable, and a user-defined number of genes containing the most spatial information. The results from both techniques achieve high performance when reconstructing spatial information in *D. melanogaster* and also generalize to zebrafish (*Danio rerio*). Furthermore, we identified novel *D. melanogaster* genes that carry important positional information and were not previously suspected. We also show how the indirect use of the full datasets’ information can lead to data leakage and generate bias in overestimating the model’s performance. Lastly, we discuss the applicability of our approaches to other feature selection problems outside the realm of single-cell sequencing and the importance of being able to handle probabilistic training labels. Our source code and detailed documentation are available at https://github.com/TJU-CMC-Org/SingleCell-DREAM/.

## Introduction

Single-cell RNA sequencing (scRNA-seq) has been rapidly gaining popularity and allows biologists to gain knowledge about the abundance of genes for thousands of cells, individually, from a given tissue. Such an approach does not suffer from the drawback of standard approaches where the aggregation of a large starting population of cells obscures the ability to detect cell-to-cell variation. Unfortunately, scRNA-seq approaches do not typically maintain the spatial arrangement of the cells (Karaiskos et al., 2017). Several strategies have been suggested to tackle this problem. One approach is a to employ a reference atlas in combination with the scRNA-seq data at hand (Satija et al., 2015; Karaiskos et al., 2017). The reference atlas is a database that contains the expression of genes across the tissue or model organism of interest. An example of a reference database is the Berkeley Drosophila Transcription Network Project (BDTNP) (Fowlkes et al., 2008), which is used in this study, and includes the *in situ* hybridization of 84 genes (herein referred to as “*inSitu* genes”) across the *Drosophila* embryo. In Karaiskos et al. (2017), BDTNP in combination with scRNA-seq data was used to map the cells of a *Drosophila* embryo to their three-dimensional (3-D) location, resulting in DistMap, an R package that automates the computations. Even though DistMap tackled this task effectively, it lacks the ability to identify the top *inSitu* genes containing the most spatial information or to identify genes that are not available in the *in situ* data and contain spatial information.

For more than a decade, the Dialogue on Reverse Engineering Assessment and Methods (DREAM) (Stolovitzky et al., 2007) initiative has driven crowd-sourced open science scientific contests in different areas of biology and medicine. Recently, the DREAM Single-Cell Transcriptomics Challenge (Tanevski et al., 2020), in which we participated, focused on tackling the reconstruction of the 3-D arrangement of cells using predefined number of genes. Specifically, the goal of this DREAM challenge was to use *Drosophila melanogaster* embryo as a model system and seek to determine whether one can reconstruct the spatial arrangement of cells from a stage 6 embryo by using only a limited number of genes. The challenge piggy-backed off previously published scRNA-seq datasets and a computational mapping strategy called DistMap, which leveraged *in situ* hybridization data from 84 genes of the BDTNP, which was shown to uniquely classify almost every position of the *D. melanogaster* embryo (Karaiskos et al., 2017). Of these 84 genes (herein referred to as “*inSitu* genes”) and without using hybridization data, the participants were asked to identify the most informative 60, 40, and 20 genes for subchallenges #1, #2, and #3, respectively. In addition to gene selection, each subchallenge also required participants to submit 10 locations predictions (X, Y, Z coordinates) for each of the cells using only the selected genes (Tanevski et al., 2020).

In order to identify the most informative genes, we describe two independent feature selection strategies. The first, which we named Lasso.TopX, leverages linear models using the least absolute shrinkage and selection operator (Lasso) (Tibshirani, 1996; Friedman et al., 2010) and ranking statistics. Lasso has a few important characteristics that made it desirable to use. Specifically, the models are easy to interpret because each feature gets assigned a coefficient, and the coefficients are combined linearly. It is also useful for dimensionality reduction because the resulting coefficients can be exactly zero, essentially eliminating features (James et al., 2013; Friedman et al., 2010). Our second feature selection strategy leverages deep neural networks (NN). NNs are making major advances in problem solving by allowing computers to better discover structure in high-dimensional data (LeCun et al., 2015). By linking multiple non-linear layers together, we sought to use deep learning in order to discover subsets of genes that would not have otherwise been possible with more traditional linear approaches.

In what follows, we describe our techniques and the novel elements that allowed us to meet the objectives of the DREAM challenge. Notably, Lasso.TopX allows a user to specify the exact number of key features they are interested in. And to take advantage of DistMap’s probabilistic mapping where a cell’s location is not always unique, we also describe how NNs can be trained using weak supervision (Zhou, 2018) for use in linear regression. Importantly, although not an objective of the DREAM challenge, we extend our techniques to other genes by looking for non-*inSitu* genes that also carry spatial information.

## Methods

In summary, we used two methodologies to identify the most informative features (*D. melanogaster* genes): an approach based on deep NN models and an approach based on Lasso models and ranking statistics, which we call Lasso.TopX. Both are supervised approaches that use training data. We then utilized inference techniques on the trained models to obtain a list of the most important 60/40/20 *inSitu* genes. In order to help baseline our results prior to the end of the competition, we also leveraged a process (herein named Random) that picked genes randomly. For the selected genes using NN, Lasso.TopX, and Random, we passed only those genes into DistMap (Karaiskos et al., 2017) to obtain the spatial predictions.

### Data Made Available by Competition Organizers

Below is a summary of the data provided to us by the DREAM challenge:

• *Reference database:* The reference database comes from the expression patterns (Fowlkes et al., 2008) of the *in situ* hybridizations of 84 genes from the BDTNP project. The *in situ* expression of 84 genes is quantified across the 3,039 *D. melanogaster* embryonic locations.
• *Spatial coordinates:* X, Y, and Z coordinates were supplied for the 3,039 locations of the *D. melanogaster* embryo (Karaiskos et al., 2017).
• *scRNA-seq:* Three expression tables were provided; the raw, normalized, and binarized expression of 8,924 genes across 1,297 cells (Karaiskos et al., 2017).
• *DistMap source code* was provided, and it was used to identify the cell locations in the initial publication (Karaiskos et al., 2017).
• *Zebrafish data:* In order to test the generalizability of our techniques outside of *D. melanogaster*, we applied them to zebrafish embryos using scRNAseq datasets from Satija et al. (2015) and *in situ* hybridizations from the ZFIN collection (Howe et al., 2013) as further described in Tanevski et al. (2020).

### Cell Locations

For each cell (*n* = 1,297) available in the RNA sequencing data, we generated training labels representing their 3-D positions by running DistMap (Karaiskos et al., 2017) with the following inputs:

- scRNA-seq expression data, both raw and normalized
- the Reference database
- the spatial coordinates

Briefly, DistMap calculates several parameters, a quantile value and one threshold per *inSitu* gene, to predict the cell locations. It employs these values to binarize the expression of the genes’ and calculate the Matthews correlation coefficients (MCC) for every cell-bin (embryo location) combination. By doing this, DistMap maps a cell to multiple likely positions. Lasso.TopX and NN approaches (described below) use these MCC values to determine the training labels.

### Feature Selection Approaches

#### Random

As a baseline approach, among the 84 *inSitu* genes from which we were allowed to pick, we randomly selected 60, 40, and 20 of them for the respective subchallenges. This random selection allowed us to benchmark (see section “Results”) how Lasso.TopX and NN feature selection approaches compared against a random process. We performed this selection step 10 times, one for each outer cross-validation (CV) fold, see *Postchallenge Outer Cross-Validation*. The importance of this comparison is to evaluate if the cost of building a method, both timewise and computationally, has any advantage over a simple approach, that does not leverage machine learning (Karathanasis et al., 2014).

#### Lasso.TopX

This approach is implemented in the R programming language and leverages the glmnet package (Friedman et al., 2010) to build generalized linear models with Lasso (Tibshirani, 1996) and ranking statistics in the final feature selection step. Lasso.TopX allows for the identification of the most informative N features, where N is 60, 40, and 20 for subchallenges 1, 2, and 3 respectively.

##### Preprocessing

We used the following data to identify the most important genes:

- *scRNA-seq*: We subset the provided normalized single-cell RNAseq dataset to include only the 84 *in situ* genes.
- *Top cell locations*: For training labels, we identified the locations of the cells using DistMap with the code provided from the challenge’s organizers, see *Cell Locations* above. For each cell, we use the bin (embryo locations) corresponding to the maximum MCC. In our feature selection process, we employed only the cells that are mapped uniquely to one location (1,010 out of 1,297 cells), Supplementary Figure S1.

##### Training flow and feature selection

**FIGURE 1:**
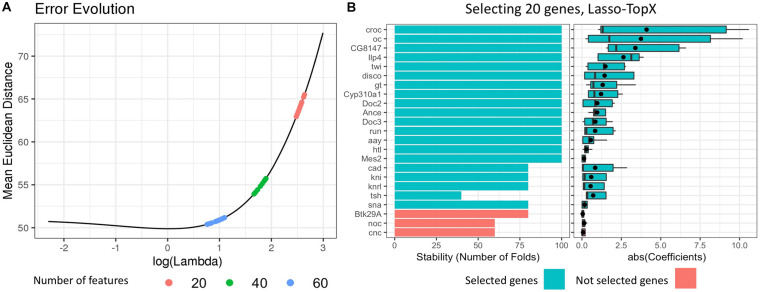
Selecting 20 genes, Lasso.TopX. **(A)** Lambda selection: the mean Euclidean distance error across the repeated fivefold CV in relation to the natural logarithm of lambda is presented for one out of 10 outer CV runs. Several models with 20 features (red dots) are produced with their lambda values ranging from 14.00083, log(14.00083) = 2.64, to 11.93777, log(11.93777) = 2.48. The lambda that produced the models with the minimum mean Euclidean distance error was selected. The same process was followed for both 40 (green dots) and 60 (light blue dots) features. **(B, Left)** Stability, the number of times a feature was selected as important across the repeated cross-validation procedure. Twenty genes are shown in one out of the 10 outer cross-validation runs. Each gene was selected at least one time by the best performing models during the repeated cross-validation (lambda = 11.93777). Twenty genes with the higher RankSum statistic were selected (blue), the last three genes (red), had the lowest RankSum statistic and left out of the final list. **(B, right)** The distribution of the absolute value of the coefficients of the selected genes. The mean value of the coefficients is shown with a black point character.

We performed the following steps to identify the important features employing Lasso.TopX:

(1) In order to identify the most important 60/40/20 features, we performed a repeated fivefold CV process. The CV was repeated 20 times for 300 different values of lambda. Lambda is Lasso’s hyperparameter, which the user needs to optimize. Intuitively, fewer features will be selected as lambda increases. The range of the lambda values was defined manually, using 70% of the data and only one time, in order for models with 60/40/20 features to be produced. In relation to the competition, we retrieved lambda ranges from glmnet packages, 100 values, and tripled the density to include 300 values. In total, we fitted 5 ^∗^ 20 ^∗^ 300 = 30,000 models. Importantly, in order to avoid overfitting, during each CV fold only the training data corresponding to this fold are standardized, and the resulting model is applied to the test data (Friedman et al., 2010). Lasso was used in the multiple Gaussian family mode, which can deal with multiple outputs, in order to make predictions for each of the X, Y, and Z locations.

(2) For each model, we extracted the following information:
  (a) *The error from the model*: The Euclidean distance of the predicted XYZ location to the top location.
  (b) The number of features that were used and their corresponding coefficients.

(3) We selected the *best lambda* value by calculating the mean error per lambda across the repeated fivefold CV (Figure 1). The *best lambda* was producing the minimum mean error and models with the desired number of features. We retained only the models corresponding to this lambda value. One lambda value and 100 models (fivefold CV ^∗^ 20 times) were selected per subchallenge.

(4) For each one of the selected models, we extracted their features and calculated two metrics.
(a) Stability: The number of times a feature was selected as important across the repeated CV procedure (Figure 1B, left, for subchallenge 3), and
(b) Mean coefficient: The mean value of the coefficients that a feature was assigned across all coordinates (Figure 1B, right), for subchallenge 3.
(5) Finally, we utilized the RankSum statistic to combine these two metrics and calculate the overall importance of the selected features. In cases where we had more than the desired number of features in our final feature list, we kept the features with the higher RankSum statistic (Figure 1). Having more than the desired number of features is possible as models with the same lambda may select different features during the repetitive CV process.

#### NN-Based Approach Using Weak Supervision

In this approach, we perform weakly supervised learning (Zhou, 2018) using NNs. After training the models, we calculate variable importance (VIP) scores to rank each gene. We describe several techniques that we used to help eliminate overfitting and make sure our model generalizes well. Because the training labels were not given to us directly and because we could not assume the max MCC from DistMap was always correct, we devised a technique that is able to use multiple training labels for the same set of input neuron values.

##### Preprocessing

All genes (*n* = 8,924) from the normalized RNAseq dataset, “dge_normalized.txt,” were used as predictor variables. For generating the training labels for the 1,297 cell locations, we used the MCC-based procedure also used by Lasso.TopX, but with one modification. Instead of using only the location (X, Y, Z) from the max MCC score, we used all locations that had an MCC score greater or equal than 95% of the max MCC score.

##### NN architecture and loss function

Model training and inference were all performed using Python 3.5, the PyTorch (Paszke et al., 2017) machine learning library, and the numpy and pandas packages. We used a fully connected NN architecture described below.

• *Input neurons*: One input neuron per RNAseq gene.
• *Hidden layers*: Two hidden layers were used, each with 100 neurons.
• *Output neurons*: Three output neurons were used (X position, Y position, and Z position).
• *Loss function*: Euclidean loss using all three output neurons.
• *Activation function*: Rectified linear units (Hahnioser et al., 2000; Nair and Geoffrey, 2017).
• *Optimizer*: Adadelta (an adaptive learning rate method) was used for gradient descent (Zeiler, 2012).
• *Regularization*: To help avoid overfitting and allow the model to generalize better, we used Hinton Dropout which can be seen as a stochastic regularization technique (Srivastava et al., 2014), set at 10% for both the hidden and input layers. While we did not utilize it, additional regularization (weight decay, L2 penalty) can be set when initializing the Adadelta optimizer.

##### Training flow

Using the preprocessed data, we performed fivefold CV 40 times for a total of 200 models. Before training, the genes were first standardized to have a mean of 0 and unit variance. For each model and to prevent bias (Ambroise and McLachlan, 2002), the parameters used for this adjustment were determined from the training splits only and then applied to the validation split.

Similarly, using the training splits only to avoid selection bias (Ambroise and McLachlan, 2002; Smialowski et al., 2009), we removed correlated genes by first identifying genes that (1) were not an *inSitu*-gene and (2) had a Pearson correlation with at least one *inSitu*-gene of ≥0.6 or ≤–0.6. We then removed these RNAseq genes from all splits. This allowed us to remove non-*inSitu* genes that might prevent VIP scores of *inSitu* genes from showing up, which we thought might be helpful because the subchallenges only asked us to report *inSitu* genes.

For every CV fold, we made sure that a cell’s gene expression values were never found in both the training and validation splits. During training, minibatch sizes of 100 were used. To help prevent the model from overfitting on the training data, we also performed early stopping by stopping the training process once the Euclidian loss on the validation fold did not improve after 50 epochs and keeping the model with the lowest loss. Because looking at the validation fold’s loss function could potentially overestimate a model’s performance, we evaluated our subchallenge scores on an external hold-out set from a separate outer CV fold (see *Results*).

##### Weak supervision enablement

There are two points mentioned previously that are important for enabling weak supervision in this workflow. We highlight them here:

- Our preprocessing step preserves all cell locations that had an MCC score greater than or equal to 95% of the max MCC score. More specifically, this means that cells that had more than one designated location were represented in the training dataset in multiple rows/observations, with identical observed input variables (gene expression) but differing observed output variables (locations).
- During the training flow, we made sure the training and validation partitions never contained information from the same cell, even if the locations were different. We accomplished this by splitting on cell names versus the row indices.

The results attributed to these decisions are further outlined in *Considerations for Weak Supervision*.

##### Feature selection

Variable importance scores were calculated and ranked for each of the 200 NN models used in the training process. We implemented (and include in our source code) Gedeon’s method (Gedeon, 1997) to come up with VIP scores for each model. For each model, we only kept the genes with the highest 60/40/20 VIP scores depending on the subchallenge. We then sorted the lists by consensus vote to obtain one list per subchallenge. For the subchallenges, only *inSitu* genes were selected.

### Location Predictions

The following steps were used by Lasso.TopX, NN, and Random approaches in order to predict 10 locations per cell:

In order to prevent overfitting, DistMap parameters were estimated using the following:

(1) Only the cells belonging in the training sets
(2) Only the genes selected during the feature selection stage, which also did not use information from the test sets

The resulting DistMap parameters were then used to binarize the expression data of the cells belonging in the test set. Finally, similar to *Cell Locations* above, for the cells in the test set, we calculated the MCC for every cell-bin combination, and we selected the 10 bins that correspond to the top 10 highest MCC scores.

### Postchallenge Outer CV

In the postchallenge phase, the organizers split the data in 10 folds, on which our approaches were rerun for stability and overfitting evaluation (Tanevski et al., 2020). Separately for each of the 10 iterations, only the respective nine training folds were used for feature selection and to train DistMap. The cells’ locations were predicted for the remaining validation fold. We refer to this postchallenge CV as the “outer CV” because any CVs described in our feature selection approaches occurred using data only within the training-folds of this outer CV.

### Blind Evaluation Metric

Prior to the competition ending, in which contestants did not have access or insight into the challenge organizers’ scoring functions, we evaluated our location predictions by calculating for each cell the mean Euclidean distance of the top 10 predicted locations from the cell location with the maximum MCC (*MeanEuclDistPerCell*). For the cells that did not map uniquely, we used the first bin among the ties as returned by R.

Then, we calculated the mean of the MeanEuclDistPerCell per outer CV fold across all cells, which we refer to as *MeanEuclDistPerFold*. Finally, the mean of the *MeanEuclDistPerFold* across all 10 outer CV folds was calculated and referred to as *MeanEuclDistAllFold*.

## Results

### Challenge Submission (Lasso.TopX or NN)

We ran both the Lasso.TopX and NN approaches for all three subchallenges. Because the challenges final scoring algorithms were not available to any participants until after the competition concluded, we compared our two feature selection approaches using a blind evaluation metric (see section “Methods”) we devised and thought might be a proxy to a good leadership score. Because the teams were allowed only one final submission to each subchallenge, we used this blind metric to determine if the results from either NN or Lasso.TopX would be submitted to each subchallenge. This blind metric was calculated individually for both feature selection techniques and for each subchallenge. Our evaluation metric suggested that Lasso.TopX may perform slightly better than NN for some subchallenges (data not shown). Based on this, our final submission used results based on NN for subchallenge 2 and Lasso.TopX for the other two. Our submitted results ranked 10th, 6th, and 4th in the three subchallenges, respectively, among ∼40 participating teams (Tanevski et al., 2020).

### Evaluation Postchallenge

**FIGURE 2:**
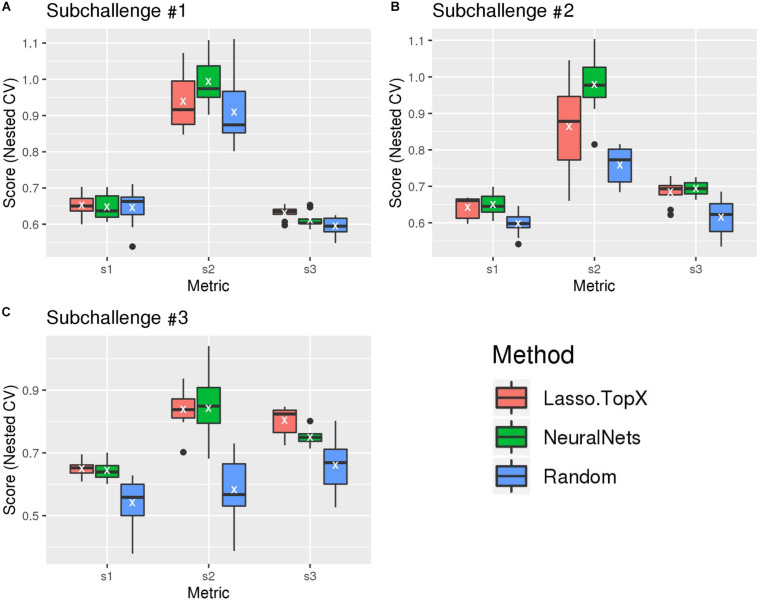
Comparison of different methods, organizers scoring functions. **(A)** For subchallenge 1, Lasso.TopX performed better than NeuralNets for s1 and s3. **(B)** For subchallenge 2, NeuralNets performed better for all scores, and for subchallenge 3 **(C)**, Lasso.TopX performed better for s1 and s3 scores. In all subchallenges, both methods performed better than random. White Xs correspond to the mean value of the respective distribution.

After the challenge ended, the organizers devised a postchallenge CV scheme [see section “Methods” and Tanevski et al. (2020) for more detail] to evaluate the robustness of the methods. It was only after this resubmission phase did the organizers make the true scoring functions (“s1,” “s2,” and “s3” scores) publicly available. Figure 2 and Supplementary Figure S2 show the results of our blind, s1, s2, and s3 metrics across the outer 10-fold CV. As expected, both Lasso.TopX and NN behaved better than Random. The results of the three true scoring schemes (Figure 2) are consistent with our previous findings using our blind metric and in agreement with the challenge paper (Tanevski et al., 2020) show that our scores have little variability and that our approaches generalize well. We also note that as illustrated by a *t*-test analysis, Supplementary Table S1, none of the observed differences between Lasso.TopX and NeuralNets are statistically significant apart from: subchallenge #1, s3, subchallenge #2 s2, and subchallenge #3 s3. The different behavior across metrics is expected as they measure different aspects of a solution as described in Tanevski et al. (2020). Because of the higher s2 score in subchallenge #1 for NN (Figure 2), we note the possibility that our NN approach could have ranked more favorably in subchallenge #1 when compared to the submitted Lasso.TopX predictions.

Additionally, in order to test the generalizability of our approaches, we applied them on a dataset generated from a different species, zebrafish. As further described in Tanevski et al. (2020), our solutions successfully reconstructed the locations of cells from a zebrafish embryo.

### Considerations for Weak Supervision

When generating training labels during the preprocessing step of NNs (see section “Methods”), there were several reasons why we allowed multiple training labels for the same cell. First, it allowed all locations of the 287 cells (Supplementary Figure S1) that did not uniquely map to be used during training. Also, we could not be certain that the max MCC was always the right value to use and wanted to better leverage the probabilistic mapping strategy enabled by DistMap.

**FIGURE 3:**
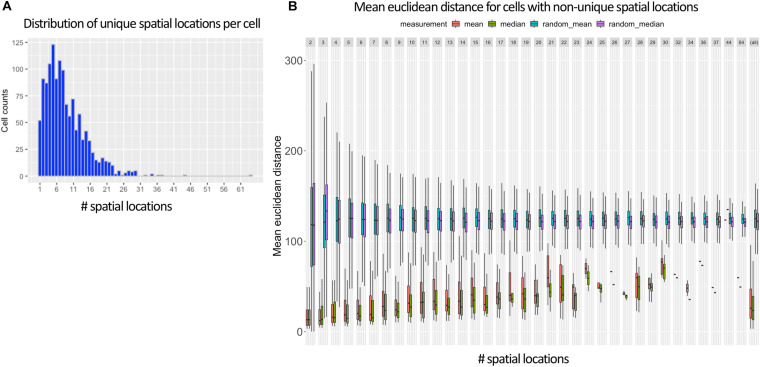
Ambiguous labels leveraged in NN approach. **(A)** Shows that most *Drosophila* cells contain more than one spatial location (mean of 7) when using DistMap’s predictions and thresholding at 95% of the max MCC. **(B)** Shows the mean Euclidean distance between the cells with non-unique training labels that meet the same 95% threshold. Cells that are selected at this threshold have mean Euclidean distances much lower than random and highlight the probabilistic nature of the training data.

Figure 3A shows that in the vast majority of the time, there exists more than one spatial location for a cell when using the 95% cutoff. The most common number of selected training labels per cell location is 5, with a mean of 7. When allowing multiple training labels per cell, our dataset became much larger: 11,491 observations instead of only 1,297 observations when only the value with the max MCC was used. One pleasant consequence of having more training data is that it makes it harder to overfit an NN, which is especially problematic in high-dimensionality settings (Bellman, 1961; Verleysen et al., 2003).

In Figure 3B, we also show that the surviving training labels per cell generally represent similar spatial coordinates when compared with randomly shuffled locations in the training data. This suggested that allowing multiple training labels per cell during training could guide the model to generalize to less-specific spatial regions without being pegged to any one location that could have been incorrectly classified.

Importantly, as mentioned in section “Methods,” when generating training and validation partitions, we made sure that a cell’s gene expression values were never found in both sets. We accomplished this by splitting on cell names versus the row indices. This is especially important because the preprocessing steps allowed the same cell to be found multiple times but with different training labels (Figure 3A). We found that if we did not split this way that we would overfit, have indirect data leakage (Luo et al., 2016), and significantly overestimate the performance of our models because the validation split could contain identical predictor variables (gene expression levels) as the training splits but with training labels that had similar (though not identical) spatial locations (Figure 3B).

### Measuring and Avoiding Data Leakage During Location Prediction

We also sought to determine what our scores would have looked like if data leakage occurred during the location prediction stage. In machine learning and statistics, data leakage can lead to inflated performance estimates when data from the validation or test set are used during training (Luo et al., 2016). Overfitting because of data leakage would have been easy to do by mistake because the provided binarized expression data, generated by DistMap, were produced using all expression data and consequently should never be used at any step of training or testing. For example, one might think that instead of modifying DistMap to perform the two-step approach described in section “Methods,” a contestant could have used the provided binarized data to directly calculate the MCC scores and the 10 cell positions. However, as is evident from Supplementary Figures S2, S3, this will lead to overestimation of performance irrespective of the scoring functions (blind, s1, s2, s3) for all the solutions (NNs, Lasso.TopX, Random) used. In both figures, we present bars and boxplots, which correspond to the overfitted location predictions using the unmodified and provided binarized data (extension “PB”) and compare it to the approach we used (extension “selGenes”).

### *InSitu* Genes With Spatial Information

**FIGURE 4:**
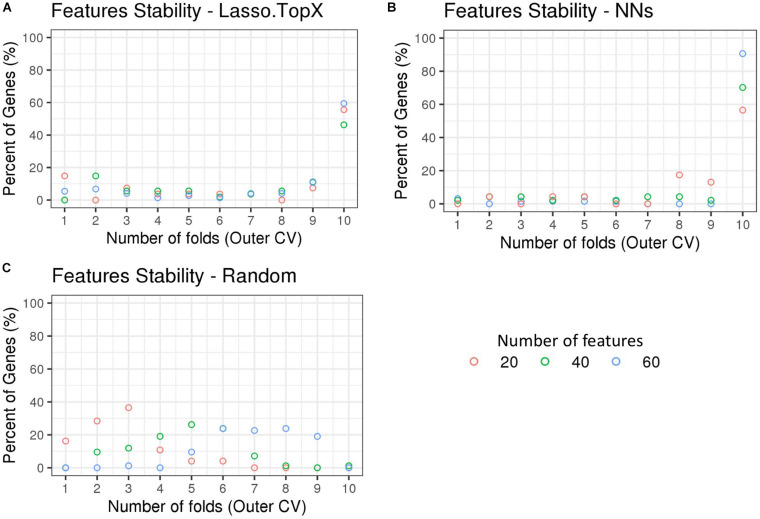
Feature stability in Nested CV using *inSitu* genes. The percentage of common selected genes across the 10-fold nested cross-validation is shown. **(A)** In the Lasso.TopX case, 27 genes were selected across all folds for subchallenge 3, red dots. Four genes or 15% percent were selected in only onefold; two genes or 7.4% percent were selected by threefold, etc. Fifteen or 56% percent were selected in all 10-fold. **(B)** Similarly for the NNs case, out of a total of 47 genes selected across all folds for subchallenge 2, green dots; 33 genes or 70% percent were selected across all 10-fold of the nested cross validation. **(C)** In the random case, 74 genes were selected across all folds for subchallenge 3, red dots. Twelve genes or 16.2% percent were selected in only one fold; 21 genes or 28.4% were selected by twofold, etc. Importantly, for all subchallenges, 0 genes were selected in all 10-fold.

We observed that the genes selected across the outer 10 CV folds were stable (Supplementary Table S2). More specifically, in the Lasso.TopX case, 74, 54, and 27 genes were selected in total for subchallenges 1, 2, and 3, respectively, with 44 (60%), 25 (46%), and 15 (56%) of them to be selected across all folds, Figure 4A. Similarly, in the NNs case, 64, 47, and 23 genes were selected, with 58 (91%), 33 (70%), and 13 (57%) of them to be selected across all folds, for subchallenges 1, 2, and 3, respectively (Figure 4B). As expected, Random did not show the same trend (Figure 4C) with zero genes selected across all folds. We observed agreement in the *in situ* genes selection between our two distinct feature selection strategies despite differences in preprocessing, features used during training, and inference models. Specifically, we observed a mean of 80, 76, and 64% agreement for subchallenges 1, 2, and 3, respectively, across the outer CV folds (Supplementary Figure S4).

### Non-*InSitu* Genes With Spatial Information

**FIGURE 5:**
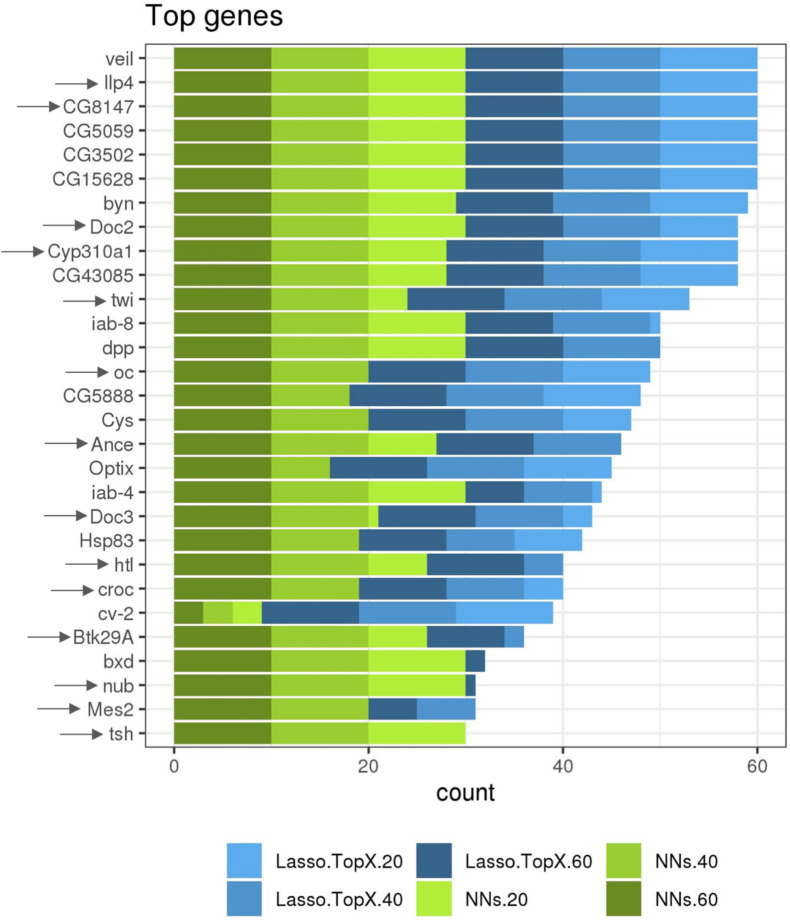
More frequently identified genes. Several *inSitu* and non-*inSitu* genes were selected across most subchallenges, methods, and cross-validation folds. Twenty-nine genes, 14 *inSitu* (arrow) and 15 non-*inSitu*, were selected in at least 30 of a total of 60 feature selection runs, x axis. Lasso.TopX and NNs are shown in shades of blue and green, respectively. Dark, regular, and light shades correspond to selecting 60, 40, and 20 genes, respectively.

While not a focus on this competition, we additionally ran both our approaches using RNASeq data information from both the non-*inSitu* and *inSitu* genes, and we were able to discover many informative non-*inSitu* genes that also contain positional information. The list of 20/40/60 genes using the NN approach would have been composed of 56, 50, and 52%, respectively, of non-*inSitu* genes (Supplementary Table S2). In the Lasso.TopX case, 67, 66, and 70% of the selected genes were non-inSitus when selecting the most informative 20/40/60 genes (Supplementary Table S2). Similar to the *inSitu* genes analysis, we calculated the stability of the selected genes across the outer CV folds. In the Lasso case, 36.6, 28.7, and 23.7% of genes were selected across all folds of the outer CV, when selecting for 20, 40, and 60 genes, respectively (Supplementary Figure S5a). In the NNs case, 46, 59, and 67% of genes were selected across all folds when selecting 20, 40, and 60 genes, respectively, Supplementary Figure S5b. Furthermore, we observed that on average 52, 57, and 51% of genes identified by NN and Lasso.TopX were in common across the 10-fold outer CV folds, when selecting 60, 40, and 20 genes, respectively (Supplementary Figure S4b). Interestingly, we observed (Figure 5) that several non-*inSitu* genes were selected consistently across all 60 feature selection runs (60 = 2 techniques ^∗^ 3 subchallenges ^∗^ 10 outer CV folds). Specifically, 142 genes were identified across all runs consisting of 38 inSitus and 104 non-inSitus. As expected, due to the fact that *inSitu* genes contain spatial information, they were selected on average more often, 25 times out of 60, than non-inSitus, 14 out of 60. However, focusing on the most stable genes, genes that were selected in at least 30 out of the 60 runs, 15 out of 29 are non-inSitus (Figure 5).

## Discussion

Here in we presented a detailed description of our techniques and solutions we submitted in the DREAM Single-Cell Transcriptomics Challenge (Tanevski et al., 2020), namely, Lasso.TopX and NN, where we successfully identify genes containing spatial information from single-cell transcriptomics data in both *D. melanogaster* and zebrafish (*Danio rerio*).

A typical Lasso workflow consists of first identifying the best lambda, using CV, and then employing that lambda to train on the full dataset to identify the most informative features (James et al., 2013). Importantly, however, this process does not allow the user to specify a discrete number of features they are interested in because the selected lambda is not tied to a user-defined number of features (Friedman et al., 2010). Also, running the typical workflow multiples times could lead to slightly different optimal lambda values as the data splits during the CV could differ, and thus, this could lead to slightly different features and different number of features. Another approach that could be employed to meet the subchallenge requirements is to have performed the typical workflow and then select the top 20/40/60 genes from the resulting list of genes. This approach is suboptimal; for example, if someone could select the best 20 features, these 20 features would not necessarily be a subset of the best 40 features (James et al., 2013). Considering the above, we developed Lasso.TopX, which leverages Lasso and is able to identify the most important user-defined number of features, employing repeated CV to make the results less dependent on any particular choice of data split. Lasso.TopX can be applied to classification or regression problems where finding important, stable, and user-defined number of features is important.

We also note that Lasso.TopX will provide the most value if the user-defined number of features is fewer than what the traditional Lasso workflow would have chosen. Taking as an example, Figure 1A, we see that Lasso’s error is decreasing in the beginning as we move to higher values of lambda (from left to right), and then there is a local minimum [close to log(lambda) = 0], and then the Lasso error increases again. At the local minimum, Lasso provides the best features given its underlying assumptions. We suggest the user to run regular Lasso, to identify Lasso’s optimal performance point and then to define the desired number of features on the right-hand side of that point (higher lambda values).

For our NN approach, we show that a cell’s training labels do not have to be unique. This is especially useful to take advantage of training data generated from DistMap’s probabilistic mapping output. We demonstrate how to properly split training and validation data when non-unique (Figure 3A) but correlated (Figure 3B) training labels are used in order to prevent data leakage. We hope that our approach will be helpful in the active research field of weak supervision (Zhou, 2018) and as probabilistic training labels become more commonplace. A lot of the research in weak supervision for NNs has focused only on logistic regression. We hope that our findings extend the research of weak supervision to linear regression and to the genetics domain.

In relation to the stability of the *InSitu* genes’ selected across the outer 10 CV folds, Figure 4, we observed that both approaches presented similar behavior for subchallenge 3 (20 features). For the other two subchallenges NNs feature stability was higher than Lasso.TopX’s. We believe that this is due to the following reasons. Lasso’s L1 regularization will remove, somewhat arbitrarily depending on the data splits (Friedman et al., 2010), highly correlated variables from the final model. Lasso is not a greedy method; a model with more variables will not necessarily include all variables of a model with fewer variables. Our NN method took a greedy approach in which features from subchallenge 3 are a subset of those from subchallenge 2. Similarly, the features from subchallenge 2 are a subset of those from subchallenge 1.

Not all decisions were consistent between Lasso-TopX and NN feature-selection approaches. For instance, the number of features (no. of genes from RNASeq data used) and training labels (max MCC vs. 95%) used during training differ between the approaches. Therefore, differences in performance (Figure 2 and Supplementary Figures S2, S3, and Supplementary Table S1) and feature stability (Figure 4 and Supplementary Figure S5) also reflect various decisions made during the preprocessing stages. We also suspect that NN’s performance did not more substantially separate, Supplementary Table S1, from our Lasso model because the organizers’ scores (s1, s2, and s3) were based on ground truth values that used only the most probable locations and the organizers’ discarded cells in which the MCCs tied (Tanevski et al., 2020).

Lastly, while identifying non-*InSitu* genes was not a focus of the competition, we show that our approaches were able to identify non-*InSitu* genes that also contain spatial information. We show that the Lasso.TopX and NN approaches both reported similar genes. Surprisingly, when focusing on the most stable genes, slightly more than half (15 or 29) were non-*InSitu* genes (Figure 5 and Supplementary Table S2). We believe that these *D. melanogaster* genes would be good candidates for exploring in future work involving spatial information. We would like to note that these techniques are extendable to other regression or classification problems and could benefit the scientific community outside of scRNA-seq applications.

## Data Availability Statement

Publicly available datasets were analyzed in this study. This data can be found here: https://github.com/dream-sctc/Data.

## Author Contributions

PL and NK contributed equally to the design and implementation of the methods and writing the manuscript. Both authors read and approved the final manuscript.

## Conflict of Interest

The authors declare that the research was conducted in the absence of any commercial or financial relationships that could be construed as a potential conflict of interest.

## Abbreviations

DREAM: Dialogue on Reverse Engineering Assessment and Methods
scRNA-seq: single-cell RNA sequencing
Lasso: least absolute shrinkage and selection operator
NN: neural networks
BDTNP: Berkeley Drosophila Transcription Network Project
MCC: Matthews correlation coefficients
CV: cross validation
VIP: variable importance
ZFIN: The Zebrafish Model Organism Database.
